# Impact of post-ruminally infused macronutrients on bovine mammary gland expression of genes involved in fatty acid synthesis, energy metabolism, and protein synthesis measured in RNA isolated from milk fat

**DOI:** 10.1186/s40104-020-00456-z

**Published:** 2020-05-20

**Authors:** Kelly Nichols, André Bannink, Jurgen van Baal, Jan Dijkstra

**Affiliations:** 1 Animal Nutrition Group, Wageningen University and Research, PO Box 338, 6700 AH Wageningen, the Netherlands; 2 Wageningen Livestock Research, Wageningen University and Research, PO Box 338, 6700 AH Wageningen, the Netherlands

**Keywords:** Cytoplasmic crescent, Endoplasmic reticulum biogenesis, Mammary cell, Milk fat globule, Milk synthesis, Tricarboxylic acid cycle

## Abstract

**Background:**

Characterising the regulation of milk component synthesis in response to macronutrient supply is critical for understanding the implications of nutritional interventions on milk production. Gene expression in mammary gland secretory cells was measured using RNA isolated from milk fat globules from 6 Holstein-Friesian cows receiving 5-d abomasal infusions of saline, essential amino acids (AA), or glucose (GG) or palm olein (LG) without (LAA) or with (HAA) essential AA, according to a 6 × 6 Latin square design. RNA was isolated from milk fat samples collected on d 5 of infusion and subjected to real-time quantitative PCR. We hypothesised that mRNA expression of genes involved in *de novo* milk fatty acid (FA) synthesis would be differently affected by GG and LG, and that expression of genes regulating transfer of tricarboxylic acid cycle intermediates would increase at the HAA level. We also hypothesised that the HAA level would affect genes regulating endoplasmic reticulum (ER) homeostasis but would not affect genes related to the mechanistic target of rapamycin complex 1 (mTORC1) or the integrated stress response (ISR) network.

**Results:**

Infusion of GG did not affect *de novo* milk FA yield but decreased expression of FA synthase (*FASN*). Infusion of LG decreased *de novo* FA yield and tended to decrease expression of acetyl-CoA carboxylase 1 (*ACC1*). The HAA level increased both *de novo* FA yield and expression of *ACC1*, and tended to decrease expression of mitochondrial phosphoenolpyruvate carboxykinase (*PCK2*). mRNA expression of mTORC1 signaling participants was not affected by GG, LG, or AA level. Expression of the ε subunit of the ISR constituent eukaryotic translation initiation factor 2B (*EIF2B5*) tended to increase at the HAA level, but only in the presence of LG. X-box binding protein 1 (*XBP1*) mRNA was activated in response to LG and the HAA level.

**Conclusions:**

Results show that expression of genes involved in *de novo* FA synthesis responded to glucogenic, lipogenic, and aminogenic substrates, whereas genes regulating intermediate flux through the tricarboxylic acid cycle were not majorly affected. Results also suggest that after 5 d of AA supplementation, milk protein synthesis is supported by enhanced ER biogenesis instead of signaling through the mTORC1 or ISR networks.

## Background

Lactating mammary gland secretory cells respond to glucose, fatty acids (FA), and amino acids (AA) through regulated catabolic and anabolic pathways facilitating biosynthesis of milk lactose, fat, and protein. Lactose synthesis is the major anabolic fate of glucose in bovine mammary cells, while glucose oxidation generates energy for FA and protein biosynthesis [1, 2]. The glucose moiety of lactose arises entirely from intramammary glucose, but hexose phosphate intermediates can contribute to the galactose moiety of lactose [3, 4]. Carbon skeletons of catabolised intramammary AA contribute as intermediates in the tricarboxylic acid (TCA) cycle, with fates including non-essential AA (NEAA) synthesis, FA synthesis, hexoneogenesis, and oxidation [5, 6]. Acetate is the primary precursor for *de novo* FA synthesis in ruminant mammary glands, but presence of long-chain FA (LCFA) for mammary uptake can downregulate this pathway in favour of incorporation of preformed LCFA into milk fat [7, 8]. Intersections between glycolysis, hexoneogenesis, AA catabolism, protein synthesis, and lipogenesis allow mammary cells to be flexible in their use of available substrates in response to the nutritional status of the animal. However, expression of genes related to carbon transfer and energy generation in bovine mammary secretory cells have gone largely unexplored *in vivo*, particularly in response to changes in energy and protein supply.

Understanding regulation of milk protein synthesis is crucial when aiming to optimize absorptive protein supply and AA profile in dairy cattle diets to minimize excess AA catabolism and nitrogen (N) excretion [9–12]. Intracellular signaling cascades of the mechanistic target of rapamycin complex 1 (mTORC1) and the integrated stress response (ISR) have been implicated in regulation of protein synthesis in dairy cattle mammary glands during postruminal infusions of starch, glucose, casein, or AA mixtures lasting up to 36 h [13, 14]. However, several multi-day (≥ 5 d) studies with glucose and essential AA (EAA) infusions have revealed that signaling through these networks, assessed at the level of protein phosphorylation [10, 11, 15] or mRNA expression [16], does not entirely account for persistent milk protein production responses to extra energy and AA supply. Instead, studies of mRNA expression of constituent proteins of the unfolded-protein response (UPR) in mammary glands of dairy cattle suggest that endoplasmic reticulum (ER) biogenesis and secretory cell differentiation could be activated in response to 5-d increases in protein supply [16, 17]. The UPR defines a 3-arm signaling cascade that copes with perturbations in ER homeostasis by regulating protein synthesis rate, stimulating ER biogenesis, and initiating cell apoptosis [18].

We recently reported results on milk production and composition and mammary gland metabolite utilization from cows post-ruminally supplemented for 5 d with glucose or palm olein (major FA constituents are palmitic, oleic, and linoleic acid) at low and high metabolizable protein (MP) levels (created through supplementation of EAA in a casein profile) [19, 20]. Independent of MP level, glucose infusion stimulated body energy retention and reduced milk energy output, increased milk N efficiency, reduced arterial concentration of branched-chain AA, Arg, and Lys, and increased plasma flow to the mammary gland. Palm olein infusion increased milk energy output independent of MP level, did not affect body energy retention, plasma flow to the mammary gland, and milk N efficiency, and did not elicit any major effects on mammary gland AA metabolism. The high MP level increased milk energy output, did not affect body energy retention and plasma flow to the mammary gland, decreased milk N efficiency, and increased arterial concentration and mammary gland uptake of EAA. Considering these responses in milk production and mammary gland AA utilization in response to glucose, palm olein, and EAA, we investigated mammary gland expression of select genes involved in *de novo* FA synthesis, the TCA cycle, protein synthesis, and the UPR using RNA isolated from milk fat as a source of genetic material from mammary secretory cells. We hypothesised that after 5 d, mRNA expression of genes involved in *de novo* FA synthesis would be differently affected by glucose and palm olein, and that mRNA expression of proteins regulating transfer of TCA cycle intermediates would increase in response to the infused EAA. Furthermore, we hypothesised that EAA infusion would affect genes related to ER homeostasis but would not affect genes related to mTORC1 signaling or the ISR network.

## Methods

### Experimental design

Details of the experimental design, animal housing, ration composition and preparation, and feed chemical analyses have been described by Nichols et al. [19]. Briefly, 6 rumen-fistulated, second lactation Holstein-Friesian dairy cows (97 ± 13 d in milk) were randomly assigned to a 6 × 6 Latin square design to test the effects of energy from glucogenic (GG) or lipogenic (LG) substrates at low (LAA) and high (HAA) EAA levels. Individual cows were housed in tie stalls within identical climate respiration chambers, and were allowed 5 d of adaptation to the housing environment before the first experimental period began. Each experimental period consisted of 5 d of continuous abomasal infusion followed by 2 d of no infusion. Cows were fed a total mixed ration (14% crude protein) consisting of 42% corn silage, 31% grass silage, and 27% concentrate on a dry matter basis, formulated to meet 100% and 83% of net energy (NE_L_) and MP requirements for lactation [21], respectively. Daily feed intake of individual cows during the entire experiment was restricted by 10% based on their daily individual ad libitum intake measured during an adaptation period. Fresh feed was allocated twice daily, with the exception of a 34-h window over d 4 and 5 of each period where feed was dispensed in equal portions every 2 h to promote metabolic steady-state conditions in preparation for a blood sampling protocol described by Nichols et al. [20]. Cows were milked twice daily at 05:30 and 15:30 h and milk volume was recorded by weight at each milking time. Sampling and analytical procedures for milk fat, FA, protein, and lactose composition have been described by Nichols et al. [19].

Infusion lines were placed in the abomasum via the rumen cannula 7 d before the first experimental period and were checked daily for patency and position. The 6 abomasal infusion treatments were: 1) 0.9% saline (LAA-C; 90% NE_L_, 75% MP), 2) 1319 g/d glucose (LAA-GG; 100% NE_L_, 75% MP), 3) 676 g/d palm olein (LAA-LG; 100% NE_L_, 75% MP), 4) 844 g/d of a complete EAA mixture in the same profile and amount as found in casein (HAA-C; 100% NE_L_, 120% MP), 5) 1319 g/d glucose + 844 g/d EAA (HAA-GG; 110% NE_L_, 120% MP), and 6) 676 g/d palm olein + 844 g/d EAA (HAA-LG; 110% NE_L_, 120% MP), where MP and NE_L_ reflect the proportion of requirements met by the restricted feeding level of the diet plus the infusate, and are expressed relative to target requirements for cows consuming 20 kg dry matter/d and producing 30 kg/d of milk containing 40 g/kg fat and 34 g/kg protein. Treatment solutions (prepared in 10-L batches) were infused via multi-channel peristaltic pumps at a rate of 6.95 mL/min continuously for 5 d. Daily infusion dosages (g/d) of LAA-GG, LAA-LG, and HAA-C were designed to be isoenergetic based on the gross energy content of the EAA infusion. Details of the composition of the EAA mixture and palm olein FA composition are described by Nichols et al. [19].

### Milk fat collection, RNA extraction, and real-time quantitative PCR

Procedures for milk fat collection, RNA extraction, and real-time quantitative PCR were performed according to those described by Nichols et al. [17], with some modifications. During morning milking on d 5 of each period, 10 mL of milk was collected by hand from individual cows within 10 min from the time the milking machine was removed. This approach allowed each sample to be processed individually, thus minimizing the amount of time between alveolar ejection of fresh milk and placing the collected milk fat into RNase inhibitor (TRIzol Reagent; Invitrogen, ThermoFisher Scientific, Waltham, MA, USA). Samples were immediately centrifuged at 2000×*g* for 10 min at room temperature to facilitate separation of the fat fraction. This centrifugation process of whole milk is expected to render the contribution of other cellular material (immune cells, sloughed epithelial cells, stem cells) negligible in the milk fat layer [22, 23]. Approximately 1 g of the supernatant cream layer was transferred into 6 mL TRIzol Reagent, mixed vigorously, snap frozen in liquid N_2_, and stored at − 80 °C until RNA extraction. Total RNA was isolated from milk fat according to TRIzol manufacturer’s instructions for handling samples with a high fat content. Total RNA concentrations and purity were determined by optical density measurement using a Nano-Drop ND-1000 (ThermoFisher Scientific). The average 260/280 absorbance ratio of total RNA samples was adequate at 1.89 ± 0.117 (mean ± SD) and the average RNA concentration was 115 ± 64.1 ng/μL (mean ± SD). The mean RNA integrity number (RIN) was 4.5 ± 2.30 (mean ± SD). The low concentration of RNA found with this procedure may have impacted RIN measurement [24]. This RIN is low by conventional standards [25], but was consistent across all samples, suggesting it was inherent to the sample type and not due to degradation of only a few samples. An aliquot of 100 ng total RNA was reverse transcribed in a total volume of 20 μL with Superscript III (100 U; ThermoFisher Scientific) in the presence of random hexamers (250 ng; Roche, Almere, the Netherlands), dithiothreitol (0.5 mmol/L; ThermoFisher Scientific) and dNTPs (0.5 mmol/L; Roche) at 25 °C for 5 min and 50 °C for 1 h. After inactivation of the enzyme (70 °C, 15 min), cDNA was stored at − 80 °C until further analysis.

Specific primers (Eurogentec, Maastricht, the Netherlands) were intron-spanning and designed to yield amplicons in the range of 100 and 180 bp (Table 1) with efficiencies of 90–100%. Templates were amplified after a preincubation of 10 min at 95 °C, followed by amplification for 40 cycles (10 s at 95 °C, 5 s at 60 °C, and 5 s at 72 °C) on a 7500 Fast Real-Time PCR System (Applied Biosystems Deutschland GmbH, Darmstadt, Germany) by using the SensiMix SYBR Low-ROX kit (Bioline UK Ltd., London, UK). All melting curves confirmed that a single amplicon was produced. As a control, a standard cDNA was included on each qPCR plate, resulting in a single and similar amplicon for the standard and cDNA samples as evidenced by melting curve analysis. As internal standards, expression of housekeeping genes glyceraldehyde 3-phosphate dehydrogenase (*GAPDH*), ribosomal protein S9 (*RPS9*), and ubiquitously expressed transcript isoform 2 (*UXT*) were analysed. NormFinder [26] identified *RPS9* as the most stable gene across experimental period and treatment. Fold changes in gene expression relative to LAA-C were calculated by the 2^−^^ΔΔCt^ method [27] using *RPS9* expression as the reference gene.

**Table 1:** Primer sequences used for real-time quantitative PCR

Gene^1^	Forward primer (5′→3′)	Reverse primer (5′→3′)	Accession No. reference
*ACC1*	GTGAAGTTCCCTCAGGCTCTTAATC	TGTCTGAGCAGATATCCACTTCC	NM_174224.2
*CASP3*	CAGCGTCGTAGCTGAACGTA	GTTTGCTGCATCCACGTCTG	NM_001077840
*CCND1*	GCTCCTGTGCTGCGAGATG	GCTCTTTTTCACGGGCTCCA	NM_001046273
*CDC42BPA*	ATGAAAAGGATGCACGAGGGT	GGCATATCTGTTGCTCGGGT	NM_001192937.2
*CSN2*	CAGGCCTTTCTGCTGTACCA	CAAAAGTGAGGAGGGGGCAT	KC993858
*DDIT3*	CAAAGCCGGAACCTGAGGAG	TCCTGCAGGTCCTCATACCA	NM_001078163
*DDIT4*	AGCCTTTGGGATCGCTTCTC	GCTGATTTGGGGTGGGAGTT	NM_001075922
*EIF2A*	TACAGAAACCATGCCCATCA	TGCAAGTTCGGTCTCATCTG	NM_175813
*EIF2AK3*	GGCTGAAAGATGACAGCACA	AGAACTGGCTCTCGGATGAA	NM_001098086
*EIF2B5*	ACTGACAAAGGCCAGCAGTT	GACGGTGGTCACTCATCCTT	XM_002684878
*EIF4E*	CAGTGCTGTGCCTTATTGGA	TGCATGGGACTGATAACCAA	NM_174310
*FASN*	CCAAGTCGAACATGGGACAT	GATCTTGGGGTTTGGGTTGT	NM_001012669.1
*GAPDH*	GGGTCATCATCTCTGCACCT	GGTCATAAGTCCCTCCACGA	NM_001034034
*HSPA5*	TGAACGACCCCTGACGAAAG	TGCGCTCCTTGAGCTTTTTG	NM_001075148
*IDH1*	ACACTGAGTGACTGTGTGCTC	CTTGGTGACCTGGTCGTTGG	NM_181012.3
*LALBA*	AGTCCTTTCGTCCCAGCACTA	AACCGGAGTCTGCTTGATGA	JN258330
*ME2*	GTTCTCCCCGGTCAGTCTCCT	TTTTCTCACCCCGCTTCTTGC	NM_001076814.1
*MYC*	GTAGTAATTCCAGCGAGAGGCA	TAGGCTAGCTCGGCTCTTCC	NM_001046074
*PCK2*	GCATCCCAACTCTCGCTTTTG	GGGGGACTCCTTTGGGTCT	NM_001205594.1
*PPP1R15A*	CAACCAGGAGACACAGAGGA	ACTCTGGGTTGAAGGGAGG	NM_001046178
*PRKAA1*	AGCCCTTCCTTCTCTTGCTC	AGGATGCCTGAAAAGCTTGA	NM_001109802
*RPL15*	ACACTATTGGTGGCTCTCGC	ACAAACATCACGTGTTAGCGG	XM_005226176
*RPS6*	TGTGCGAAAGCCCCTAAACA	GGAGTCACGAGACGCTGAAT	NM_001015548
*RPS6KB1*	TGACAGCCCAGATGACTCAG	TGGGCTGCCAATAATCTTC	NM_205816
*RPS9*	CTGAAGCTGATCGGCGAGTA	GGGTCTTTCTCATCCAGCGT	NM_001101152.2
*UXT*	CGCTACGAGGCTTTCATCTC	CCGAGTGGTTAGCTTCCTGG	NM_001037471.2
*XBP1*s	TGCTGAGTCCGCAGCAGGTG	AGAATGCCCAACAGGATGTC	BC102639
*XBP1*u	AGACTACGTGCACCTCTGCAG	AGAATGCCCAACAGGATGTC	BC102639

^1^*ACC1* = acetyl-CoA carboxylase 1; *CASP3* = caspase 3; *CCND1* = cyclin D1; *CDC42BPA* = CDC42 binding protein kinase alpha; *CSN2* = β-casein; *DDIT3* = DNA damage-inducible transcript 3; *DDIT4* = DNA damage inducible transcript 4; *EIF2A* = eukaryotic initiation factor 2, α subunit; *EIF2AK3* = eukaryotic translation initiation factor 2, alpha kinase 3; *EIF2B5* = eukaryotic translation initiation factor 2B, ε subunit; *EIF4E* = eukaryotic translation initiation factor 4E; *FASN* = fatty acid synthase; *GAPDH* = glyceraldehyde 3-phosphate dehydrogenase; *HSPA5* = H3 histone, family 3A; *IDH1* = isocitrate dehydrogenase 1, cytosolic; *LALBA* = α-lactalbumin; *ME2* = malic enzyme 2, mitochondrial; *MYC* = MYC proto-oncogene; *PCK2* = phosphoenolpyruvate carboxykinase 2, mitochondrial; *PPP1R15A* = protein kinase AMP-activated catalytic, α1 subunit; *PRKAA1* = protein kinase AMP-activated catalytic, α1 subunit; *RPL15* = ribosomal protein L15; *RPS6* = ribosomal protein S6; *RPS6KB1* = ribosomal protein S6 kinase B1; *RPS9* = ribosomal protein S9; *UXT* = ubiquitously expressed prefoldin like chaperone; *XBP1*s = X-box binding protein 1, spliced; *XBP1*u = X-box binding protein 1, unspliced

### Statistical analysis

Variances in milk and component production and gene expression data were analysed using the MIXED procedure of SAS (SAS Institute Inc., Cary, NC). The model contained main and interaction effects of infusion treatment factors (GG, LG, and AA level) and experimental period as fixed effects and cow as a random effect. No carryover effects on milk and milk component production were observed between periods, as assessed by testing for an effect of the previous treatment in the ANOVA. Differences were considered significant at *P* ≤ 0.05 and tendencies at 0.05 < *P* ≤ 0.10. Multiple comparisons between treatment means were made using the Tukey-Kramer method when GG × AA or LG × AA interactions were detected at *P* ≤ 0.10.

## Results

### Production of milk protein, fat, lactose, and fatty acids

Infusing GG or LG at the HAA level did not affect yield of total milk or milk components differently than at the LAA level (no significant GG × AA or LG × AA interactions; *P* > 0.28; Table 2). Total milk yield was unaffected by GG and LG (*P* > 0.60) and increased at the HAA level (*P* < 0.01). Milk fat yield decreased in response to GG (*P* < 0.01), increased in response to LG (*P* < 0.01), and increased at the HAA level (*P* = 0.02). *De novo* FA (< 16 carbons) yield decreased in response to LG (*P* = 0.02), increased at the HAA level (*P* < 0.01), and was unaffected by GG (*P* = 0.88). Yield of preformed FA (> 16 carbons) decreased in response to GG (*P* < 0.01), increased in response to LG (*P* < 0.01), and was not affected by AA level (*P* = 0.25). Yield of mixed FA (16 carbons) was not affected by GG, LG, or AA level (*P* ≥ 0.14). Yields of milk protein and lactose were unaffected by GG and LG and increased at the HAA level (*P* < 0.01).

**Table 2:** Milk and component yield (kg/d) from dairy cows receiving abomasal infusions of saline (control; C), glucose (GG), and palm olein (LG) at low (LAA) and high (HAA) essential amino acids levels for 5 d^1^

	Treatment^2^						SEM	*P*-value^3^				
	LAA-C	LAA-GG	LAA-LG	HAA-C	HAA-GG	HAA-LG		GG	LG	AA	GG × AA	LG × AA
Total milk	25.1	24.9	24.5	28.6	29.5	28.8	1.58	0.61	0.69	< 0.01	0.37	0.54
Fat	1.09	0.96	1.23	1.16	1.04	1.30	0.053	< 0.01	< 0.01	0.02	0.94	0.98
Fatty acids^4^												
*De novo*	0.28	0.28	0.26	0.32	0.32	0.30	0.018	0.88	0.02	< 0.01	0.68	0.77
Preformed	0.38	0.25	0.51	0.40	0.26	0.53	0.025	< 0.01	< 0.01	0.25	0.72	0.98
Mixed	0.36	0.37	0.38	0.37	0.39	0.39	0.022	0.36	0.14	0.51	0.78	0.83
Protein	0.79	0.78	0.75	0.98	1.02	0.97	0.047	0.70	0.28	< 0.01	0.29	0.51
Lactose	1.18	1.18	1.16	1.30	1.34	1.31	0.075	0.54	0.79	< 0.01	0.59	0.62

^1^Data are least squares means from the final 3 d of infusion (see Nichols et al. [19] for further details)

^2^LAA-C, 0.9% saline; LAA-GG, 1319 g/d glucose; LAA-LG, 676 g/d palm olein; HAA-C, 844 g/d of a complete EAA mixture in the same profile and amount as found in 1.5 kg casein; HAA-GG, 1319 g/d glucose + 844 g/d EAA; HAA-LG, 676 g/d palm olein + 844 g/d EAA. Infusions of LAA-GG, LAA-LG, and HAA-C supplied 20.5 MJ/d, and HAA-GG and HAA-LG supplied 41.0 MJ/d. For all treatments *n* = 6

^3^GG = effect of energy from glucose; LG = effect of energy from fat; AA = effect of protein level

^4^Calculated using milk fat yield and individual milk fatty acid (FA) weight proportions presented by Nichols et al. [19] to determine yield on a mass basis using the average proportion of FA in milk (93.7 g/100 g) derived from individual FA molecular weight and corrected for the glycerol portion of triacyglycerides and other milk lipid classes according to Glasser et al. [28]. *De novo*-synthesized FA (< 16 carbons) originate from mammary *de novo* synthesis, performed FA (> 16 carbons) originate from mammary plasma extraction, mixed FA (16 carbons) originate from both *de novo*-synthesized and preformed FA

### Expression of *CSN2* and genes related to fatty acid synthesis and energy-yielding pathways

Infusing GG or LG at the HAA level did not affect the expression of *CSN2* or genes associated with energy-yielding pathways differently than at the LAA level (no GG × AA or LG × AA interactions; *P* ≥ 0.17; Table 3). Infusion of GG decreased *CSN2* and *FASN* expression 11% and 38%, respectively, compared with infusions with no GG (*P* = 0.02). Infusion of LG tended to decrease *ACC1* expression (*P* = 0.08), and decreased *CSN2* expression 22% compared with infusions with no LG (*P* < 0.01). The HAA level increased *ACC1* expression 76% over infusions at the LAA level (*P* = 0.01), and tended to decrease *PCK2* expression (*P* = 0.06).

**Table 3:** Mammary gland expression (arbitrary units) of milk protein genes, and genes regulating milk fatty acid synthesis and energy-yielding pathways in dairy cows receiving abomasal infusions of saline (control; C), glucose (GG), and palm olein (LG) at low (LAA) and high (HAA) essential amino acid levels for 5 d^1^

	Treatment^2^						SEM	*P*-value^3^				
	LAA-C	LAA-GG	LAA-LG	HAA-C	HAA-GG	HAA-LG		GG	LG	AA	GG × AA	LG × AA
*ACC1*	1.19	1.54	0.69	2.07	2.44	1.51	0.399	0.21	0.08	0.01	0.98	0.92
*CSN2*	1.19	0.87	0.80	1.26	0.97	0.87	0.212	0.02	< 0.01	0.47	0.89	0.98
*FASN*	1.15	0.92	1.18	1.39	0.71	1.57	0.205	0.02	0.55	0.62	0.23	0.66
*IDH1*	1.21	0.94	1.42	1.41	0.95	1.79	0.300	0.16	0.25	0.46	0.73	0.73
*LALBA*	1.31	1.28	1.38	2.22	1.25	2.09	0.433	0.17	0.93	0.35	0.19	0.77
*ME2*	1.67	4.29	1.78	2.32	2.76	1.31	1.529	0.12	0.65	0.32	0.17	0.33
*PCK2*	1.16	1.05	1.23	0.96	0.72	0.60	0.339	0.46	0.55	0.06	0.79	0.37

^1^Values are least-squares means from milk fat collected on d 5 of each period. Fold changes in gene expression from RNA captured in milk fat globules relative to LAA-C were calculated by the 2^-ΔΔCt^ method after normalizing to *RPS9*

^2^LAA-C, 0.9% saline; LAA-GG, 1319 g/d glucose; LAA-LG, 676 g/d palm olein; HAA-C, 844 g/d of a complete EAA mixture in the same profile and amount as found in 1.5 kg casein; HAA-GG, 1319 g/d glucose + 844 g/d EAA; HAA-LG, 676 g/d palm olein + 844 g/d EAA. Infusions of LAA-GG, LAA-LG, and HAA-C supplied 20.5 MJ/d, and HAA-GG and HAA-LG supplied 41.0 MJ/d. for all treatments *n* = 6

^3^GG = effect of energy from glucose; LG = effect of energy from fat; AA = effect of protein level

### Expression of genes related to the mechanistic target of rapamycin complex 1 and the integrated stress response network

Expression of mTORC1- and ISR-related genes are presented in Table 4. Expression of translation initiation factor *EIF2B5* tended to be affected by a LG × AA interaction, where EIF2B5 expression was numerically higher (55%) at the HAA level than at the LAA level (*P* = 0.07). Gene expression of *EIF2A*, *EIF2AK3*, *EIF4E*, *PRKAA1* and *RPS6KB1* was not affected by treatment (*P* ≥ 0.12).

**Table 4:** Mammary gland expression (arbitrary units) of genes related to the mechanistic target of rapamycin complex 1 and the integrated stress response network in dairy cows receiving abomasal infusions of saline (control; C), glucose (GG), and palm olein (LG) at low (LAA) and high (HAA) essential amino acid levels for 5 d^1^

	Treatment^2^						SEM	*P*-value^3^				
	LAA-C	LAA-GG	LAA-LG	HAA-C	HAA-GG	HAA-LG		GG	LG	AA	GG × AA	LG × AA
*EIF2A*	1.08	0.95	1.04	1.16	0.87	1.06	0.207	0.12	0.59	0.81	0.54	0.78
*EIF2AK3*	1.09	1.05	1.25	1.66	1.54	1.39	0.240	0.70	0.80	0.15	0.85	0.31
*EIF2B5*	1.12^ab^	0.91^b^	1.04^ab^	1.04^ab^	1.07^ab^	1.61^a^	0.198	0.62	0.17	0.05	0.50	0.07
*EIF4E*	1.29	0.97	1.40	1.40	1.16	1.57	0.271	0.18	0.49	0.37	0.84	0.86
*PRKAA1*	1.33	1.83	1.54	2.04	1.94	1.64	0.399	0.64	0.56	0.89	0.34	0.28
*RPS6KB1*	1.25	0.80	0.99	0.91	0.74	0.92	0.205	0.14	0.54	0.78	0.47	0.50

^a,b^Means within a row with no common superscripts differ (*P* ≤ 0.05)

^1^Values are least-squares means from milk fat collected on d 5 of each period. Fold changes in gene expression from RNA captured in milk fat globules relative to LAA-C were calculated by the 2^-ΔΔCt^ method after normalizing to *RPS9*

^2^LAA-C, 0.9% saline; LAA-GG, 1319 g/d glucose; LAA-LG, 676 g/d palm olein; HAA-C, 844 g/d of a complete EAA mixture in the same profile and amount as found in 1.5 kg casein; HAA-GG, 1319 g/d glucose + 844 g/d EAA; HAA-LG, 676 g/d palm olein + 844 g/d EAA. Infusions of LAA-GG, LAA-LG, and HAA-C supplied 20.5 MJ/d, and HAA-GG and HAA-LG supplied 41.0 MJ/d. For all treatments *n* = 6

^3^GG = effect of energy from glucose; LG = effect of energy from fat; AA = effect of protein level

### Expression of genes related to endoplasmic reticulum homeostasis and biogenesis, and cell differentiation and turnover

Genes related to ER homeostasis and biogenesis, and cell differentiation and turnover are presented in Table 5. mRNA expression of the serine/threonine kinase *CDC42BPA* was affected by a GG × AA interaction, where expression was 40% higher with GG infusion at the LAA level compared with the HAA level (*P* = 0.05). Infusion of GG tended to increase the expression of cell growth regulator *MYC* (*P* = 0.10), and tended to decrease expression of unspliced (u) ER stress-related transcription factor *XBP1* (*P* = 0.08). Infusion of LG decreased the expression of *XBP1*u (*P* = 0.02) and increased the proportion of spliced (s) *XBP1* to *XBP1*u (*P* = 0.04). The HAA level tended to increase *MYC* expression (*P* = 0.08), and increased the expression of *XBP1*s and the proportion of *XBP1*s to *XBP1*u (*P* ≤ 0.01).

**Table 5:** Mammary gland expression (arbitrary units) of genes for endoplasmic reticulum homeostasis and biogenesis, and cell differentiation and turnover in dairy cows receiving abomasal infusions of saline (control; C), glucose (GG), and palm olein (LG) at low (LAA) and high (HAA) essential amino acid levels for 5 d^1^

	Treatment^2^						SEM	*P*-value^3^				
	LAA-C	LAA-GG	LAA-LG	HAA-C	HAA-GG	HAA-LG		GG	LG	AA	GG × AA	LG × AA
*CASP3*	1.14	0.98	0.81	1.24	1.07	1.13	0.253	0.44	0.30	0.35	0.97	0.62
*CCND1*	1.91	1.75	0.90	1.59	1.07	3.58	1.317	0.80	0.71	0.45	0.89	0.26
*CDC42BPA*	1.13^ab^	2.14^a^	1.07^b^	1.55^ab^	1.53^ab^	1.17^ab^	0.240	0.06	0.38	0.31	0.05	0.53
*DDIT3*	1.89	1.76	2.42	1.79	2.83	2.12	1.265	0.36	0.39	0.44	0.25	0.84
*DDIT4*	1.22	1.49	0.67	0.96	0.88	1.74	0.484	0.84	0.81	0.64	0.70	0.17
*HSPA5*	1.09	1.24	1.17	1.14	1.62	1.22	0.385	0.28	0.95	0.21	0.46	0.89
*MYC*	1.07	1.22	1.06	1.12	1.65	1.37	0.253	0.10	0.56	0.08	0.35	0.51
*PPP1R15A*	1.08	1.11	0.95	1.13	0.97	1.04	0.185	0.60	0.40	0.85	0.44	0.89
*RPS6*	1.10	1.63	0.97	1.29	1.41	0.78	0.197	0.14	0.14	0.35	0.34	0.38
*RPL15*	1.08	1.11	0.99	1.13	0.97	1.04	0.190	0.61	0.50	0.74	0.46	0.98
*XBP1*												
Spliced	1.03	0.80	0.91	1.45	1.47	1.23	0.162	0.50	0.26	< 0.01	0.41	0.74
Unspliced	1.02	0.80	0.78	1.25	0.96	0.79	0.118	0.08	0.02	0.57	0.81	0.45
Spliced/unspliced	1.03	1.10	1.25	1.22	1.56	1.77	0.168	0.26	0.04	0.01	0.45	0.36

^a,b^Means within a row with no common superscripts differ (*P* ≤ 0.05)

^1^Values are least-squares means from milk fat collected on d 5 of each period. Fold changes in gene expression from RNA captured in milk fat globules relative to LAA-C were calculated by the 2^-ΔΔCt^ method after normalizing to *RPS9*

^2^LAA-C, 0.9% saline; LAA-GG, 1319 g/d glucose; LAA-LG, 676 g/d palm olein; HAA-C, 844 g/d of a complete EAA mixture in the same profile and amount as found in 1.5 kg casein; HAA-GG, 1319 g/d glucose + 844 g/d EAA; HAA-LG, 676 g/d palm olein + 844 g/d EAA. Infusions of LAA-GG, LAA-LG, and HAA-C supplied 20.5 MJ/d, and HAA-GG and HAA-LG supplied 41.0 MJ/d. For all treatments *n* = 6

^3^GG = effect of energy from glucose; LG = effect of energy from fat; AA = effect of protein level

## Discussion

### Milk fat RNA technique

RNA isolated from milk fat was used as the source of genetic material from mammary secretory cells to measure gene expression in this study. A small fraction of cytoplasmic material from mammary secretory cells is entrapped during secretion of milk fat globules [29, 30]. Milk fat globules are an unconventional non-tissue source of extracellular mRNA [31], but representation of the mammary secretory cell transcriptome in the genomic material found in cytoplasmic crescents of milk fat globules has been validated in primates [23, 32] and ruminants [33, 34]. Cytoplasmic crescents in bovine milk are small and are present in a low proportion of milk fat globules [29]. However, the relatively high fat content of ruminant milk allows the abundance of cytoplasmic material in milk fat to be adequate for RNA isolation [33, 35]. Compared with the common technique of mammary tissue biopsy, milk fat collection to obtain RNA from secretory cells for *in vivo* signaling analysis is noninvasive and enables a larger number of animals to be sampled at a higher frequency, and with relative ease compared to tissue biopsy. In this way, the technique allows mRNA expression of genes related to mammary gland metabolism to be characterized under a variety of experimental conditions. RNA isolated from milk fat has been previously used to assess the effects of nutritional intervention on mammary cell metabolic pathways in studies with dairy cattle [17, 35]. We employed this technique in the current study to assess changes in mRNA expression of a select set of genes related to FA synthesis, energy metabolism, and protein synthesis in response to increased absorptive supply of glucose, palm olein, and EAA. The genes of interest were selected based on results of previous investigations in response to energy and protein supply using RNA isolated from biopsied mammary tissue [16] and milk fat globules [17], and identification of their associated pathways via microarray analysis [36].

### Fatty acid synthesis

Acetyl-CoA carboxylase 1 (ACC1), encoded by *ACC1*, and fatty acid synthase (FAS), encoded by *FASN*, are key participants in *de novo* biosynthesis of milk lipids in bovine mammary cells. The first step of *de novo* FA synthesis, the carboxylation of acetyl-CoA to form malonyl-CoA, is catalysed by ACC1 [37]. Next, FAS catalyses the condensation of malonyl-CoA and acetyl-CoA and forms FA of ≤16 carbons [38]. Coordinated regulation of these enzymes in dairy cattle mammary glands has been predominantly investigated under conditions of milk fat depression, a phenotype characterised by reduced yield of *de novo*-synthesized FA (mainly < 16 carbon FA) and total milk fat [39, 40]. In the current study, GG infusion did not affect *de novo* FA yield or *ACC1* expression, but decreased *FASN* expression by 38%. Considering the key role of FAS in *de novo* FA synthesis, reduced *FASN* expression does not align with our observation of no change in *de novo* FA yield. However, GG infusion decreased total milk fat yield by 16%. This was driven by the stimulatory effect of increased circulating insulin on lipogenesis in extra-mammary tissues, leading to the reduction of circulating LCFA (> 16 carbons) for incorporation into milk fat [19, 20]. Lipogenic genes in bovine mammary cells are under the control of transcription factor families, such as the sterol regulatory element binding proteins (SREBP) [41, 42]. *FASN* is a transcriptional target of SREBP1 [43], and both constituents are downregulated when total milk fat yield is decreased during diet-induced milk fat depression or feed restriction [35, 44]. The relatively lower abundance of LCFA in mammary cells for incorporation into milk triacyglycerides (TAG), as suggested by reduced arterial LCFA concentration during GG infusion [19, 20], may have resulted in a coordinated downregulation of the expression of rate-limiting enzymes in the glycerol 3-phosphate pathway, which are also controlled by SREBP [43]. Taken together, regulation of FAS at the transcriptional level after 5 d of glucose infusion did not impact downstream changes required to affect *de novo* FA yield.

Infusion of LG decreased *de novo* FA yield by 8%, tended to decrease *ACC1* expression, but did not affect *FASN* expression. High intracellular concentrations of LCFA or their acyl-CoA esters inhibit ACC1 in lipogenic tissues [37, 45]. This inhibitory effect could have arisen in the presence of LCFA from palm olein (predominantly C16:0 and C18:1), and may have had a relatively larger impact on ACC1 than FAS, thus impacting their transcripts accordingly. A similar pattern was observed in response to EAA infusion, where *de novo* milk FA yield and *ACC1* expression both increased at the HAA level, but *FASN* expression was not affected. Overall, coordinated changes in mRNA expression of *ACC1* and *FASN* were not observed in response to glucose, palm olein, or EAA infusions in this study. Our results suggest that when mammary cells are supplied with glucose, palm olein, or EAA for 5 d, a distinctly coupled increase or decrease in both enzymes at the transcriptional level is not required to produce phenotypic changes in the FA composition of milk fat. In line with this observation, previous studies have observed variations between milk FA synthesis and lipogenic gene expression. In lactating cows and goats, supplements of corn oil or sunflower oil plus wheat starch reduced milk FA content and yield, but little or no variation in mRNA levels of mammary lipogenic genes was observed after 25 or 27 d of feeding [46, 47]. In the study of Invernizzi et al. [48], mammary gland expression of *ACC1* and *FASN* and *de novo* milk FA synthesis from cows was increased after 7 d of dietary supplementation with saturated fat but not fish oil. By d 21 of this supplementation, differences in gene expression between saturated fat and fish oil had disappeared but the differences in *de novo* milk FA synthesis remained.

### Tricarboxylic acid cycle

Intermediates arising from AA catabolism in mammary cells contribute to TCA cycle anaplerosis [5, 6]. The mitochondrial isoforms of malic enzyme (ME), encoded by *ME2*, and phosphoenolpyruvate carboxykinase (PEPCK), encoded by *PCK2*, channel TCA cycle intermediates derived from NEAA and certain EAA (Ile, Thr, Val) to pyruvate [49, 50]. PEPCK catalyses the GTP-driven transformation of oxaloacetate to phosphoenolpyruvate (PEP) [49, 51]. Malic enzyme converts malate to pyruvate, independent of glycolytic flux [50, 52]. The mitochondrial forms of both enzymes support oxidative flux through pyruvate. Therefore, these channels are important in mammary secretory cells where glucose is used for biosynthesis and is not the main fuel for the TCA cycle [53], and they may be upregulated when the rate of intramammary AA catabolism increases in response to MP supplementation [6]. In the current study, both the intramammary catabolism of Ile and Val and the calculated intramammary glucose deficit for lactose and fat synthesis increased in response to infused EAA at the high MP level [20]. Therefore, we hypothesised that mRNA expression of *PCK2* and *ME2* would increase, reflecting increased flux of carbon skeletons of AA (AA-C) through the TCA cycle to spare glucose in support of the increase in lactose and fat yield during EAA infusion.

In contrast to our hypothesis, *PCK2* expression tended to decrease at the HAA level, and *ME2* expression was not affected. Alternative to sparing glucose from oxidation in the TCA cycle, intracellular AA-C could support lactose synthesis through contribution to galactose synthesis [6, 20, 54]. Upregulation of the cytosolic isoform of PEPCK would favour this process over the mitochondrial form by incorporating AA-C into cytosolic PEP [6]. Cytosolic PEP can enter the pentose phosphate cycle, producing hexose phosphate intermediates contributing to galactose [3, 4], whereas AA-C incorporated into mitochondrial PEP favours NEAA synthesis and oxidation [1, 49]. Data on gene expression or enzyme activity of the cytosolic or mitochondrial isoforms of PEPCK and ME in bovine mammary glands in response to energy or protein supply are scarce. We recently reported that extra energy from protein or fat supplementation did not affect *PCK2* expression in mammary cells, and protein supplementation decreased expression of *ME2*, but only in combination with low fat diets [17]. However, such an interaction between aminogenic and lipogenic nutrient supply on *ME2* did not occur in the present experiment. Further investigation at the transcriptional and translational level of enzymes involved in intramammary carbon transfer would allow a clearer picture of energy balance and AA-C metabolism in lactating bovine mammary glands in response to glucogenic, lipogenic, and aminogenic substrates.

### Protein synthesis

Previous reports where expression of milk protein genes are not indicative of observed milk protein yields were substantiated by the current study [16, 17, 55]. Milk protein yield was not affected by GG or LG infusion, but expression of *CSN2*, encoding β-casein, decreased 11% and 22% in response to GG and LG, respectively. The HAA level increased milk protein yield 28% but did not affect *CSN2* expression. Similarly, in a previous study, the same dose and profile of abomasally infused EAA increased milk protein yield 30% and did not affect mammary *CSN2* expression [16].

As hypothesised, and in agreement with others [10, 11, 16], we observed minimal effects on mRNA expression of mTORC1 and ISR participants in response to the 5-d infusion of EAA, despite the 0.2 kg/d increase in milk protein yield at the HAA level. Expression of *EIF2B5*, encoding the ε subunit of eukaryotic translation initiation factor 2B (eIF2B), tended to increase at the HAA level, but more so in the presence of LG. eIF2B is a 5-subunit guanine nucleotide exchange factor responsible for the formation of active eIF2-GTP complexes that deliver initiator Met-tRNA to ribosomes during each round of translation initiation [56]. To our knowledge, there are no previous reports investigating mRNA expression of eIF2B in response to high fat conditions in mammary cells. In heart muscle of rats, free FA increased the nucleotide exchange activity of eIF2B [57]. Greater *EIF2B5* expression has the potential to facilitate protein translation. Although this observation is in alignment with the increase in milk protein yield at the HAA level, *EIF2B5* expression increased more at the HAA level in the presence of LG, while the response in milk protein yield was independent of LG. This, and our finding that mRNA expression of no other mTORC1 or ISR constituents were affected by GG, LG, or AA level, supports our hypothesis that other mechanisms may have greater influence on phenotypic milk protein yield responses after 5 d of nutrient infusion.

Previous investigation at the mRNA level from biopsied mammary tissue suggested that ER biogenesis in mammary cells is responsive to EAA supply, potentially through regulation on separate UPR signaling arms [16]. Initiation of the UPR in mammalian cells occurs by dissociation of the ER chaperone binding immunoglobulin protein (BiP/GRP78) onto 3 ER transmembrane proteins, namely protein kinase R-like ER kinase (PERK), activating transcription factor 6 (ATF6), and inositol-requiring enzyme 1 (IRE1), which initiate their respective UPR arms [18]. The elevated proportion of the active spliced *XBP1* available for translation relative to the inactive unspliced *XBP1* mRNA (spliced/unspliced) at the HAA level suggests induction of the IRE1-mediated arm of the UPR. Splicing of *XBP1* mRNA is needed to generate the active transcription factor (*XBP1*s), which has been implicated in lactating bovine mammary glands in response to increased protein supply [16, 17], and at the onset of lactation [58]. The translation product of *XBP1*s regulates genes involved in ER formation and expansion, and secretory vesicle maturation in a variety of differentiated secretory cells, such as antibody-secreting plasma cells [59], gastric zymogenic cells [60], pancreatic endocrine and exocrine cells [61, 62], and mammary secretory cells [63, 64].

The increased ratio of *XBP1*s to *XBP1*u in response to EAA infusion suggests potential stimulation of ER biogenesis in support of greater mammary cell protein secretory capacity, in line with our hypothesis. Similarly, Nichols et al. [16] observed an increased *XBP1*s to *XBP1*u ratio in response to 5-d abomasal EAA infusion in the same AA profile and dose, and Nichols et al. [17] observed a similar increase in response to rumen-protected protein supplementation for 27 d, but only in low-fat diets. The ratio of *XBP1*s to *XBP1*u also increased in response to LG infusion in the current study. Increased *XBP1* splicing in response to high fat diets has been reported in murine liver and skeletal muscle [65, 66]. Moreover, there could be a link between increased *XBP1* transcript splicing in response to LG and increased *EIF2B5* expression at the HAA level in the presence of LG. The IRE-1-mediated arm of the UPR is protective in its effort to regulate protein synthetic capacity of cells by reducing ER protein load and inhibiting apoptosis [18]. Likewise, activation of eIF2Bε upon toll-like receptor stimulation was protective against ER-stress-mediated cell death in macrophages [67]. Together with the increased *XBP1*s to *XBP1*u ratio, the tendency for *EIF2B5* expression to be increased relatively more at the HAA level in the presence of LG may arise from a response whereby FA promote adaptation in mammary secretory cells in support of persistent milk protein synthesis when EAA are in abundant supply. Overall, adaptive ER biogenesis in bovine mammary cells in response to EAA supplementation and the presence of LCFA, suggested here by the increased *XBP1*s to *XBP1*u ratio, is a potentially attractive explanation for observed increases in milk protein synthesis. However, continued investigation of mechanisms involving changes in rate of cell turnover, secretory cell differentiation, and secretory cell capacity under supplementation with energy and protein substrates is necessary to characterize the adaptive mechanisms of lactating mammary glands in response to nutritional interventions. In particular, the temporal characterisation of transcriptional and translational changes in participants of cell signaling networks, such as the link between mRNA expression of UPR genes and ER biogenesis, should be further elucidated in lactating bovine mammary glands from both conventional tissue samples and from application of the herein described milk fat RNA technique.

Altered secretory cell number in response to increased nutrient supply could be contributing to faster protein synthesis in response to EAA supplementation. To test this hypothesis, we analysed mRNA expression of select genes encoding proliferation and apoptosis pathways. Genes related to cytoskeletal dynamics and cell proliferation, *CDC42BPA* and *MYC*, were both affected by glucose and EAA infusion. Expression of *CDC42BPA* tended to increase 40% when GG was infused at the LAA level compared with HAA-GG. *CDC42BPA* encodes myotonic dystrophy kinase-related CDC42-binding kinase (MRCK) α, a downstream effector of the cell division control protein 42 homolog (CDC42) involved in cytoskeleton assembly and organization. This gene has been characterised in mammary cells primarily in view of breast cancer pathogenesis [68], and in cattle in association with cell reorganization during bacterial infection from *Mycobacterium avium* ssp. *paratuberculosis* [69]. The fact that no GG × AA interaction was observed for milk protein yield suggests that the increase in *CDC42BPA* expression with GG infusion at the LAA level may be unrelated to synthesis of export proteins in mammary cells. The c-Myc transcription factor, encoded by *MYC*, regulates genes required for cell proliferation, growth, differentiation, and apoptosis, but is also associated with ribosome biogenesis and protein synthesis required for cell growth [70, 71]. Expression of *MYC* tended to increase independently in response to GG and the HAA level, but milk protein yield was only affected at the HAA level. Nichols et al. [16] observed increased mammary *MYC* expression but no extra stimulation of milk protein synthesis when glucose was added to EAA infusions. In murine pancreatic islet cells, mRNA expression of *MYC* was increased *in vitro* under supraphysiological glucose concentrations [72], and during hyperglycaemia *in vivo* [73]. In hepatocytes, growth and proliferation in response to protein and energy restriction and re-feeding schemes altered expression of *MYC* as cells progressed through the development cycle [74–76]. In bovine mammary glands, *MYC* expression was associated with cell proliferation in response to milking frequency [77], and during the dry period and early lactation [78]. Taken together, these findings suggest that mammary cell hyperplasia or hypertrophy could be affected by transcription factors in response to glucogenic and aminogenic substrates, but further study is required to determine the biological importance with respect to persistent milk component synthesis throughout lactation.

## Conclusions

Expression of genes involved in *de novo* FA synthesis in mammary gland secretory cells is sensitive to 5-d infusions of glucose, palm olein, and EAA, but a coupled increase or decrease in *ACC1* and *FASN* mRNA expression was not required to produce phenotypic changes in *de novo* FA yield. Reduced *FASN* expression in response to glucose infusion may have been related to downregulation of TAG synthesis. Possible inhibition of ACC1 by LCFA during palm olein infusion may have decreased *ACC1* expression, in line with increased preformed FA in milk fat. Stimulation of *de novo* FA synthesis by EAA infusion was associated with increased *ACC1* expression. Expression of *PCK2* tended to decrease during EAA infusion, and *ME2* expression was not affected, suggesting that increased intramammary AA catabolism did not impact these enzymes at the mRNA level, or that AA-C shuttling occurred through different channels of the TCA cycle. We found no effects of 5-d glucose, palm olein, or EAA infusions on mRNA expression of participants in the mTORC1 signaling network. Expression of the ISR constituent *EIF2B5* tended to increase with EAA infusion, but only in the presence of palm olein, and this did not coincide with extra milk protein synthesis when EAA and palm olein were infused together. Instead, splicing of the *XBP1* transcript at the HAA level and the absence of an effect on *CSN2* expression suggests increased milk protein synthesis in response to EAA infusion potentially occurred through stimulation of ER biogenesis in support of greater cell secretory capacity. Palm olein infusion also increased the proportion of spliced *XBP1* relative to unspliced, but this was not associated with an increase in milk protein yield. *XBP1* splicing in response to palm olein and the tendency for *EIF2B5* expression to be increased at the HAA level only in the presence of palm olein may indicate a protective effect of FA in mammary cells in support of persistent milk protein synthesis when EAA are in abundant supply.

## Data Availability

The datasets generated and analysed during the current study are available from the corresponding author upon reasonable request.

## Abbreviations

AA: Amino acid
AA-C: Amino acid carbon skeleton
ACC1: Acetyl-CoA carboxylase 1
ATF6: Activating transcription factor 6
BiP/GRP78: Binding immunoglobulin protein
CDC42: Cell division control protein 42 homolog
EAA: Essential amino acid
eIF2B: Eukaryotic translation initiation factor 2B
ER: Endoplasmic reticulum
FA: Fatty acid
FAS: Fatty acid synthase
IRE1: Inositol-requiring enzyme 1
ISR: Integrated stress response
LCFA: Long-chain fatty acid
ME: Malic enzyme
MP: Metabolizable protein
MRCK: Myotonic dystrophy kinase-related CDC42-binding kinase
mTORC1: Mechanistic target of rapamycin complex 1
N: Nitrogen
NE_L_: Net energy for lactation
NEAA: Non-essential amino acid
PEP: Phosphoenolpyruvate
PEPCK: Phosphoenolpyruvate carboxykinase
PERK: Protein kinase R-like ER kinase
SREBP: Sterol regulatory element binding proteins
TAG: Triacylglycerides
TCA: Tricarboxylic acid cycle
UPR: Unfolded protein response
XBP1: X-box binding protein 1
